# A comparison of forensic anthropology casework across two urban Midwest Medical Examiner's offices, 2013–2022

**DOI:** 10.1111/1556-4029.70439

**Published:** 2026-08-16

**Authors:** Erin B. Waxenbaum, Lindsay H. Trammell, Anne L. Grauer

**Affiliations:** ^1^ Department of Anthropology Northwestern University Evanston Illinois USA; ^2^ St. Louis County Office of the Medical Examiner St. Louis Missouri USA; ^3^ Department of Anthropology Loyola University Chicago Chicago Illinois USA

**Keywords:** caseload, casework, Chicago, consultation, decomposition, forensic anthropology, Illinois, Missouri, month of discovery, seasonal variation, St. Louis

## Abstract

Forensic anthropology has garnered greater attention in the academic and medicolegal communities with more practitioners embedded in Medical Examiner's and Coroner's offices. Practitioners have varied roles and responsibilities, leaving few opportunities to compare the nature, breadth, and outcomes of their casework. Sharing data provides the foundations upon which resource distribution, planning, and needs assessment can be based, providing essential information for training the next generation of professionals. This study reports on casework conducted by three forensic anthropology practitioners across two Midwest Medical Examiner's jurisdictions. Between 2013 and 2022, forensic anthropologists working as consultants at the Cook County Medical Examiner's Office (CCMEO) completed 140 cases, with the St. Louis Medical Examiner's Office (SLMEO) having an embedded anthropologist completing 288 cases. The greatest number of cases were recorded in 2022 for both agencies and entered the CCMEO in June and July, and the SLMEO between October and December. Recovery of decomposed and fully skeletonized decedents from outdoor surface contexts (CCMEO) and indoor scenes (SLMEO) was most common. Biological profile (CCMEO) and radiographic identification (SLMEO) were the most requested types of evaluation. Differences exist in timing, analysis, and report submission. Variation stems from contractual differences between offices, environmental contexts, and the type of consult requested. Greater discussion and comparison of the range of services and expertise that forensic anthropologists provide broadens the understanding of the field, informs our stakeholders, allows for the formulation of best practices, and serves as data upon which decisions regarding protocol, funding, resources, and need can be based.

**Highlights**

- Between 2013 and 2022, the CCMEO completed 140 forensic anthropology consults, SLMEO completed 288.
- Peak cases were in June (CCMEO) and July, October, November, and December (SLMEO) and during the most recent year evaluated (2022).
- Decomposed and fully skeletonized with outdoor surface (CCMEO) and indoor (SLEMO) recoveries were most common.
- Biological profile (CCMEO) and radiographic identification (SLMEO) were the most common evaluations.
- Discussion of forensic anthropology services and expertise broadens our understanding of the field.

## 1 INTRODUCTION

Forensic anthropology has garnered greater attention in the academic and medicolegal communities with more practitioners embedded in Medical Examiner's and Coroner's (ME/C) offices and increased coursework and forensic‐oriented graduate programs offered at colleges and universities across the country [1, 2, 3, 4, 5]. However, practitioners situated within the medicolegal system often have varied roles and responsibilities, with few opportunities to compare the nature, breadth, and outcomes of their casework [2, 3]. Sharing data will provide foundations upon which resource distribution, planning, and needs assessment can be based and will provide essential information for training of the next generation of professionals in the field.

The present analysis compares work conducted by three forensic anthropologists (FA) across two jurisdictions: two working as part‐time consultants and one employed full‐time within a Medical Examiner's Office (MEO). In the Cook County MEO (CCMEO) in Illinois, two FAs (EBW and ALG) consult on a part‐time, case‐by‐case basis. The St. Louis County, Missouri MEO (SLMEO) employs one full‐time FA (LHT) who worked concurrently as a medicolegal death investigator during the period under analysis.

Comparison of anthropology casework from varied jurisdictions provides essential data upon which planning, needs assessment, and resource determinations can be appraised. Continued discussion and publication of casework detail and composition not only serve future practitioners within anthropology but the broader audience of forensic scientists who engage with or request our expertise.

## 2 MATERIALS AND METHODS

### 2.1 Cook County and the Cook County medical Examiner's office

Illinois supports a mixed Medical Examiner and Coroner system, with the CCMEO serving as the only Medical Examiner's office in the state. The CCMEO jurisdiction extends over 1635 square miles, of which approximately 11% (70,000 acres; 110 square miles) is designated as forest preserves. The jurisdiction also covers 63 miles of lakeshore and includes approximately 5.2 million people representing 45% of the population of Illinois [6, 7]. Approximately 16,000 deaths are reported annually, with 5600 requiring medicolegal examination and half of those examined cases are autopsied [8].

The two contracted FAs at the CCMEO (EBW, ALG) review CCMEO investigator's reports, photographs taken at the scene of discovery, upon arrival at the CCMEO, and throughout the autopsy process under the pathologists' direction, and radiographs taken of the decedent. Based on this information, they may request maceration, which is completed by Medical Examiner's staff. Additional photographs are taken by CCMEO Photography Department staff under the anthropologists' direction during anthropological analysis [8].

### 2.2 St. Louis county and the St. Louis County Medical Examiner's Office

The state of Missouri is a mixed Medical Examiner and Coroner system with three medical examiner's offices in the St. Louis Metro area. The SLMEO is located in Berkeley, Missouri, a municipality of St. Louis County. St. Louis County has an approximate population of just under 1 million people and averages around 11,000 reportable deaths annually with approximately 1750 requiring medicolegal investigation [9].

The one full time FA (LHT) at the SLMEO worked concurrently as a medicolegal death investigator during the period under investigation and subsequently worked as an FA full time (which began in January 2022, during the final year of data collection for the present analysis). The anthropologist provides consultations at the request of the forensic pathologist or a coroner in neighboring Missouri or Illinois jurisdictions. All casework analysis and maceration, if necessary, takes place at the St. Louis County MEO by the FA. Depending on the case type and FA availability, the FA responds to or participates in scene recovery in collaboration with the requesting agency. Pathology assistants coordinate postmortem radiography and photography once remains have arrived for analysis at the MEO, and the forensic anthropologist takes additional photographs and radiographs, as needed, throughout the examination process.

### 2.3 Methods of analyses

With the objective of comparing the composition of forensic anthropology casework at the CCMEO and the SLMEO between 2013 and 2022, the following variables were recorded and compared: month of decedent discovery, depositional environment, condition of remains upon initial discovery, type of anthropology consult requested, time from discovery to consultation request, time from consultation request to analysis, and time from analysis to report submission. For the purposes of this project, a “case” is defined as material presented for forensic anthropological assessment that is assigned a Medical Examiner case number and a final report is generated.

As reported previously by Waxenbaum and Grauer [8], multiple consult types were assessed by the FAs at the CCMEO. These included determination of human versus nonhuman remains, biological profile, trauma analysis, MNI determination, and other requests specifying a particular component of biological profile or assessment of anatomical consistency between human remains discovered from different points in time. For the SLMEO, the type of consult request also extends to radiographic evaluation, which was not requested of FAs at the CCMEO during the duration of this analysis. At SLMEO, with an embedded FA, nonhuman remains that are received are not regularly assigned a case number (and no report is generated), as they are immediately cleared by the FA. For the CCMEO, FA consultants may be contacted for assessment prior to a possible nonhuman case being assigned a CCMEO case number. In other circumstances, CCMEO nonhuman cases are assigned an ME case number and a report is generated.

The month of initial discovery and decedent depositional environment were appreciated at the CCMEO based on investigator's reports received with initial anthropologist consult request. At SLMEO, the determination of the depositional environment was reported directly by the FA who was often at the scene of discovery. Depositional environmental conditions included ground surface outdoors, indoors, aqueous, completely buried, partially buried, vehicular, displaced, or unknown. The displaced category included remains noted in the investigator's report or suggested by the forensic pathologists (at the CCMEO) or directly by the FA (SLMEO) to have been discovered apart from the original point of deposition.

Condition of remains upon initial discovery was assessed from photography taken at the scene and/or upon arrival of the remains at the CCMEO since the FAs are rarely called into the field to assist with recovery but was noted directly by the SLMEO forensic anthropologist at the scene or in the morgue. Categories for condition of remains upon discovery include fully fleshed (no visually apparent decompositional openings in the soft tissue), decomposing/mummified, fully skeletonized, displaying thermal alteration (some component of soft or skeletal tissue demonstrated thermal alteration), or unknown condition upon initial deposition. Similar descriptors for condition of remains were recorded at the SLMEO, with the difference that the FA had immediate and direct involvement with active cases and could directly document details.

Demographic variables of decedents, including estimation of age at death, sex, and population affinity/ancestry, are not included in this analysis, as the focus is on the composition of anthropological casework, rather than the decedents themselves. However, demographic analyses of decedents in anthropological casework in other jurisdictions have been provided by Falsetti [10], Grisbaum and Ubelaker [11], and Komar [12].

## 3 RESULTS

Between 2013 and 2022, FAs working at the CCMEO completed a total of 140 cases (averaging 14 cases per year), with the SLMEO completing 288 cases (averaging 28.8 cases per year) (Figure 1). The greatest number of cases occurred at the CCMEO in 2022 (*n* = 21) and the fewest in 2020 (*n* = 8). For SLMEO, the greatest number of cases similarly occurred in 2022 (*n* = 55) and the fewest in 2013 (*n* = 9) (Figure 1).

**FIGURE 1:**
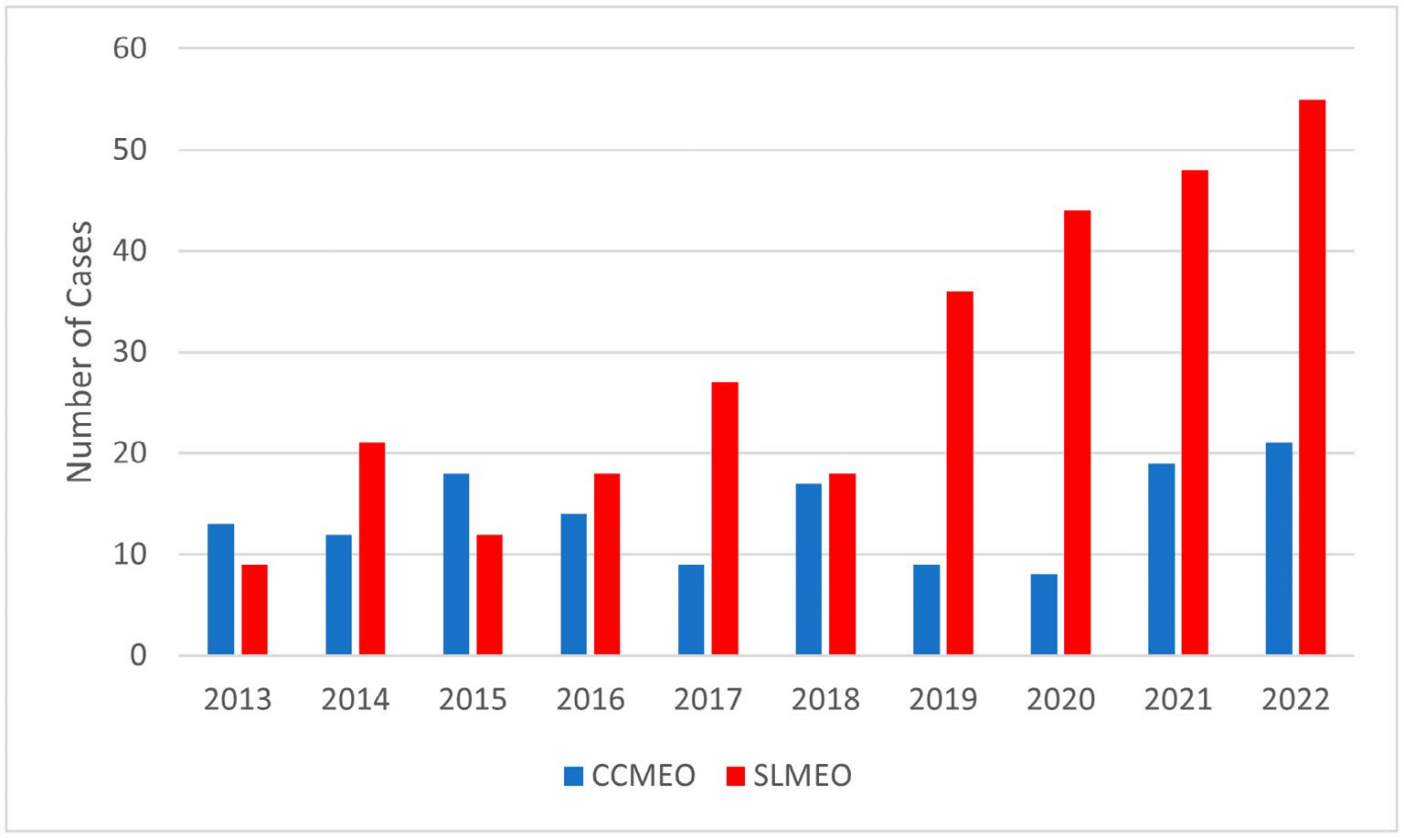
Forensic anthropology consults per year, 2013–2022 (CCMEO, mean = 14.0, total *N* = 140; SLMEO, mean = 28.8, total *N* = 288).

Casework varied by month of discovery across both jurisdictions (Figure 2). Most cases at the CCMEO were discovered in June (*n* = 22) and the fewest in December (*n* = 1), whereas at the SLMEO, the majority of cases occurred in July, October, November, and December (*n* = 27), with the fewest cases discovered in January (*n* = 12) (insert Figure 2).

**FIGURE 2:**
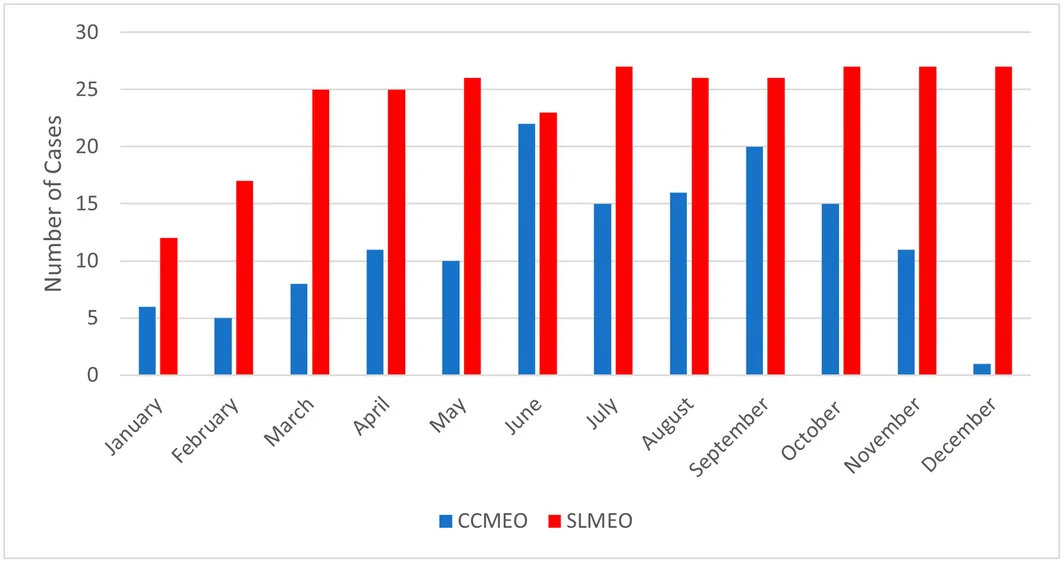
Forensic anthropology cases by month of discovery.

The discovery context of casework varied between the two jurisdictions (Table 1). However, most cases were outdoor surface recoveries (CCMEO, *n* = 39) and indoor (SLMEO, *n* = 107). The fewest cases at CCMEO were discovered in a vehicle (*n* = 4). For SLMEO, the fewest cases were appreciated in an aqueous environment or partially or completely buried (*n* = 15).

**TABLE 1:** Forensic anthropology casework by discovery context/depositional environment (CCMEO *N* = 140, mean = 14/year; SLMEO *N* = 288, mean = 28.8/year).

	CCMEO		SLMEO	
Surface	49	35%	93	32%
Indoors	36	26%	107	37%
Aqueous	16	11%	15	5%
Complete/partial buried	15	11%	15	5%
Vehicle	4	3%	42	15%
Unknown/displaced	20	14%	16	6%

The state in which the remains presented upon initial discovery also varied between jurisdictions (Table 2). However, most cases for both jurisdictions were decomposed/mummified (CCMEO = 54%, *n* = 75; SLMEO = 37%, *n* = 106), with fully skeletonized being the next most common category (CCMEO = 38%, *n* = 53; SLMEO = 30%, *n* = 85).

**TABLE 2:** State of remains for anthropology casework (CCMEO *N* = 140; SLMEO *N* = 288).

	CCMEO		SLMEO	
Fully skeletonized	53	38%	85	30%
Decomposing/mummified	75	54%	106	37%
Fully fleshed	7	5%	41	14%
Thermal damage	3	2%	56	19%
Unknown	2	1%	0	0%

While consult requests from pathologists often include multiple components (e.g., biological profile and trauma analysis; human/nonhuman, if human, biological profile) (Table 3), for the CCMEO, the majority of requests included biological profile estimation (69%, *n* = 96) and trauma analysis (50%, *n* = 70), while for the SLMEO, the majority consisted of radiographic evaluation (52%, *n* = 150) (a task assigned to pathologists at the CCMEO), trauma analysis (34%, *n* = 97), and biological profile (31%, *n* = 89).

**TABLE 3:** Casework by type of consult request (CCMEO *N* = 140; SLMEO *N* = 288).

	CCMEO*		SLMEO*	
Biological profile	96	69%	89	31%
Trauma analysis	70	50%	97	34%
Human/nonhuman	17	12%	0	0%
Min number of individuals	5	4%	0	0%
Specific feature	1	1%	0	0%
Radiographic evaluation	0	0%	150	52%
Medicolegal significance	0	0%	17	6%
Other	2	1%	12	4%

* A single consult may include multiple components resulting in percentages exceeding 100%.

While forensic anthropology consultation timelines can vary from case to case (Table 4), on average for the CCMEO, 66.8 days elapsed from discovery to anthropology consult, 59.3 days from consultation request to anthropology visit, and 13.3 days from anthropology analysis in person to report submission. For the SLMEO, 4.3 days elapsed from discovery to anthropology consult, 2.2 days from consultation request to anthropology analysis, and 7.6 days from anthropological analysis to report submission. Total time from initial discovery of remains to report submission averaged 130 days for the CCMEO and 14 days for SLMEO.

**TABLE 4:** Forensic anthropology consultation timeline between 2013 and 2022 (averaged days).

	CCMEO	SLMEO
**Time to notification**		
Mean	66.8	4.3
Range	1427	84
SD	212.5	7.5
**Time from notification to analysis**		
Mean	59.3	2.2
Range	924	29
SD	98.6	2.7
**Time from analysis to report**		
Mean	13.3	7.6
Range	82	54
SD	15.3	10.2
**Total time from discovery to report**		
Mean	130	14.0
Range	1457	154
SD	221.1	15.6

## 4 DISCUSSION

Forensic anthropology consultations at the CCMEO and SLMEO demonstrate both similarities and considerable variation between the years 2013 and 2022. Our evaluation suggests that the presence of an embedded anthropologist, professional environments, and geographic factors play an important role in shaping the casework of FAs. For instance, as noted in Figure 1, casework increased appreciably at the SLMEO over the decade under consideration, while remained fairly constant and only slightly increased at the CCMEO (except for the years affected by COVID). These patterns are understood, in part, by concerted outreach efforts by the FA (LHT) at the SLMEO to familiarize employees throughout the jurisdiction with ways in which forensic anthropology can contribute to investigations. These efforts included training of local and regional law enforcement departments, Missouri and Illinois Coroner offices, and Missouri forensic pathologists, as well as word of mouth and referrals from individuals and agencies who regularly request forensic anthropological consultation. The increased casework and consultation requests at the SLMEO led to the shift of the FA (LHT) from a dual role as a medicolegal death investigator and forensic anthropologist to the role of a full‐time FA.

At the CCMEO, while similar efforts were made, FAs serve as part‐time consultants, which greatly limits interaction between the forensic anthropologists and medical examiners, as well as with investigators. This also appears to affect the overall caseload between jurisdictions (Figure 1). Nonetheless, through invitations to fellows to observe forensic anthropology evaluations and formal and informal presentations to CCMEO pathologists and fellows, greater knowledge of the field of forensic anthropology may correlate with slight increases in consultations over the period of evaluation.

Data from the two jurisdictions indicates that geography influences multiple aspects of forensic anthropology casework. Although St. Louis County is located approximately 400 miles southwest of Cook County/Chicago, they share relatively similar northern midwestern US climatic conditions, with St. Louis recording an average summer high of 77.7°F, 33.8°F winter low, and 41.04 inches of precipitation [13] from 1990 to 2020 and Chicago recording an average summer high of 79.4°F, 24.7°F winter low, and 37.86 inches of precipitation [14] from 1991 to 2020. These similarities likely contribute to winter months (December–March) yielding fewer forensic cases discovered for both jurisdictions (Figure 2). However, it is interesting to note that forensic case discoveries remain far more constant throughout the calendar year in St. Louis than in Chicago. In part, this might be due to the SLMEO FA participating in radiographic identification/assessments and the more rural environment conducive to year‐round hunting and hiking.

Geography also influences the discovery context/depositional environment (Table 1) and the state of remains (Table 2) of cases assigned to the FA for analysis. For both the CCMEO and SLMEO, surface and indoor discovery contexts dominated the casework, with fully skeletonized and decomposing/mummified remains being the most common state of remains. The association between discovery context and state of remains is not unexpected given that depositional environment influences the state of remains [15]. However, these similarities of occurrence between the two jurisdictions mask important differences. At the CCMEO, surface and indoor discovery contexts account for 61% of all cases and at the SLMEO, these contexts account for 69%. The increased percentage of indoor cases in SLMEO is likely connected to many decedents being found within their homes and requiring comparative antemortem/postmortem identification by the FA.

The greatest differences noted between jurisdictions are seen ‘in house’; that is, substantial variation between types of consultation requested of the FAs at the CCMEO and SLMEO and the timeline to completion of reports. Pilloud and colleagues' [16] survey of forensic anthropologists' caseloads note an average of 12.33 sole‐authored skeletal analysis reports generated per year, which is below the average of both jurisdictions assessed presently. As noted by Pilloud and colleagues, averages between jurisdictions vary by employment type, including both embedded FAs in FA‐specific labs, as well as academic FAs (those primarily employed in a university setting) who consult with jurisdictions as needed. Institutions such as the Defense POW/MIA Accounting Agency (DPAA) and Harris County Institute of Forensic Sciences employ FAs that engage with a far greater volume of casework annually than academic FAs [16]. For the CCMEO and SLMEO, academic FAs (EBW and ALG) author far fewer reports than those directly employed by a MEO (LHT).

There is also significant variation in case type between jurisdictions. At the CCMEO, 69% of the consult requests included biological profile and 50% of total casework included trauma analysis. Proportionately fewer biological profile requests (31%) and trauma analysis (34%) were performed by the forensic anthropologist at the SLMEO. In part, these proportional differences may be due to delineated roles assigned to FAs within the CCMEO and SLMEO. At the SLMEO, the full‐time FA (LHT) participates in the radiographic evaluation of decedents in order to assist with identification through antemortem and postmortem comparisons (constituting 52% of consult type requested). As noted previously, at the CCMEO, forensic pathologists are responsible for this task.

Variation in casework between jurisdictions/offices has also been noted by Crowder and colleagues [2]. In their comparison of casework by full‐time forensic anthropologists working in four medical examiners offices in 2015, they note an average of 45–69% of casework involved trauma analysis, with biological profile analysis constituting only 4–15% [2]. These values are similar to trauma analysis in the present study but differ appreciably from the percentages of CCMEO and SLMEO biological profile requests, where both offices conduct a greater number of biological profile evaluations. This may be a product of the geographic and socioeconomic environments within which they work, as well as internal protocol for evaluation for unidentified decedents.

Crowder et al. [2] also include an FA casework category of “identification,” which entails individual identification through antemortem and postmortem comparisons, comprising 10–43% of total casework. While an even greater percentage of cases at the SLMEO involve radiographic identification (52%), none were requested by the CCMEO over the period of evaluation. Limited recognition of FAs skillset by ME/C offices may account for this discrepancy. Given Crowder and colleagues' emphasis on the effects of a “paradigm shift in the conceptual framework by which the forensic anthropologist operates,” which has expanded the role of FAs within medical examiner's offices [2:358–359], and concerted efforts to inform professional communities of FA skillsets at the CCMEO and SLMEO, the number and types of analyses requested of FAs will likely continue to change.

Evaluating the overall discrepancies in casework between the CCMEO and SLMEO is complicated and results from a variety of factors. For instance, varying socioeconomic conditions between the densely populated urban environment of Chicago and Cook County (5,109,292 recorded residents in 2022 [17]), and conditions in St. Louis County (990,414 recorded residents in 2022; a more mixed urban/rural environment that does not include the city of St. Louis [18]) might play a significant role. Urban density is associated with limited and/or restrictive housing, leading to high cost to rent, and conversely, to the presence of greater resources needed for support. Access to support encourages individuals and families with limited resources to migrate to cities [19]. According to a report from the US Department of Housing and Urban Development [19:1], “While both sheltered and unsheltered homeless populations have declined since 2011, in more recent years, the unsheltered homeless population has increased substantially. Unsheltered homelessness in major cities and mostly urban Continuums of Care (CoCs) rose by nearly 25 percent, from 87,345 in 2015 to 109,252 in 2017.” Specifically, according to a 2022 Chicago point‐in‐time study [20], approximately 5889 individuals were recorded as experiencing homelessness in 2016. Homelessness data for St. Louis County is less available, though an affordable housing strategic plan indicates 487 people experienced homelessness in January 2019 (e.g., [21]). The greater number of biological profile requests at the CCMEO (69% vs. 31% at the SLMEO) might be a direct result of a greater homeless population and, hence, unidentified decedents at the CCMEO for whom antemortem/postmortem comparisons are more difficult, rendering biological profile an essential tool toward identification.

Two other notable points of deviation between the CCMEO and SLMEO include requests for radiographic evaluation (0% at the CCMEO and 52% at SLMEO) and the identification of nonhuman versus human remains (12% at the CCMEO and 0% at the SLMEO) (Table 3). As mentioned previously, the stark differences in requests for radiographic evaluation is due to the employment of full‐time radiology technicians and pathologists at the CCMEO who complete the evaluations. However, importantly, it is also likely due to the limited recognition of FA skillsets to assist in this area. The other major variation, requests to assist with identification of human versus nonhuman remains is largely due to recovered bone being brought to the CCMEO and assigned a case number prior to FA consultation. However, an increasing number of law enforcement officials in Cook County and surrounding areas are contacting the Cook County Sheriff's Police and FAs directly from the scene for confirmation of identification prior to formally initiating a CCMEO case. Given the embedded employment of an FA at SLMEO, nonhuman cases are triaged and dismissed before a Medical Examiner case number is assigned.

Yet another observed difference between offices is the overall caseload of each office versus the percentage of forensic anthropological consultation requests. At the CCMEO, approximately 5600 deaths fall under the MEO jurisdiction and require further investigation (with half of those undergoing postmortem examination) [7], but on average 14 (0.005%) of those go to FA consultation. Whereas in SLMEO, approximately 1750 deaths undergo postmortem examination and 29 (1.66%) of those go to FA consult. This is likely related to the reliance on the FA at SLMEO to complete radiographic identifications and the smaller proportions of decedents whose antemortem records cannot be recovered.

Substantial variation in consultation timelines between the CCMEO and SLMEO warrants scrutiny, as these represent the greatest differences between the jurisdictions. As noted in Table 4, time from discovery to notification (request for FA evaluation) averaged over 2 months (66.8 days) at the CCMEO but less than a week at the SLMEO (4.3 days). This difference is due primarily to CCMEO casework comprising numerous cold cases and employing part‐time FA consultants, while the FA at the SLMEO worked predominately on current cases and is employed full‐time. Time from notification to analysis also varied significantly (59.3 days at the CCMEO and 2.2 days at the SLMEO). The substantial proportion of decedents found in a state of decomposition at the CCMEO (54% of cases) relied upon CCMEO staff for maceration, rather than being completed by the FA, whereas at the SLMEO, only 37% of the decedents required maceration prior to analysis, and this was performed by the full‐time employed FA.

The duration between case analysis and submission of the report demonstrated the least variation between jurisdictions (13.3 days at the CCMEO and 7.6 at the SLMEO). However, the relatively small difference may be explained by FA contractual differences at the CCMEO and the SLMEO, Part‐time FAs at the CCMEO must coordinate email communication between CCMEO pathologists, radiologists, and photographers, rather than being able to readily communicate face‐to‐face at the facility. The variation might also be explained by competing obligations and time commitments experienced by the part‐time FAs, while an embedded full‐time FA has the ability to prioritize and triage casework in step with concurrent responsibilities.

These results have important implications for Medical Examiner's offices applications for or maintenance of NAME (National Association of Medical Examiners) accreditation. The NAME accreditation checklist includes questions concerning the completion of all postmortem examinations within 60–90 calendar days from the time of autopsy [22]. The results presented here advocate for embedded FAs in ME/C offices, when possible, to decrease time to case completion [2, 23].

## 5 CONCLUSION

Data collected and evaluation of the casework conducted by FAs at the CCMEO and SLMEO provide essential insights into our field. For instance, geographic environments within which forensic anthropologists work appear to play a key role in FA caseload, since seasons influence the discovery of decedents. Socioeconomic environments also play an important role, with homelessness contributing substantially to the state of remains and types of analysis requested of FAs. Substantial differences in the timelines from discovery of decedents to submission of final reports are undeniably associated with staffing within medical examiner's offices. The presence of a full‐time, embedded FA substantially reduced the duration of case completion, a factor that might prove essential in high‐profile cases, increased numbers of cases within a jurisdiction, and/or acquiring NAME accreditation. Likewise, the availability of full‐time staff other than FAs, including forensic pathologists, radiology technicians, and photographers, has the benefit of utilizing specialist knowledge and skills but can increase the duration between decedent discovery and submission of final report.

Our data and analyses strongly suggest that internal education within Medical Examiner's and Coroner's offices, as well as clear communication between FAs and law enforcement agencies, contribute immensely to the frequency and scope of calls for forensic anthropology consultation. Acceleration and improvement in these efforts resulted in the effective use of the forensic anthropologists' skills and greater contributions to essential data needed for resolution of cases of unidentified decedents and the evaluation of ante, peri, and postmortem trauma. The presence of anthropologists “in house” substantially contributes to this effort.

The role and breadth of forensic anthropology are expanding both academically and within the medicolegal community. Greater discussion and comparison of the range of services, support, and expertise that forensic anthropologists provide ME/C offices broadens the understanding of the field, allows for the formulation of best practices, and serves as data upon which decisions regarding protocol, funding, resources, and need can be based. With additional practitioners collecting and sharing their data, a clearer window into best practices, and the scope and utility of the field will be appreciated by both our colleagues and the greater forensic scientific community.

## FUNDING INFORMATION

The authors have nothing to report.

## CONFLICT OF INTEREST STATEMENT

The authors declare no conflict of interest.

## Data Availability

The data that support the findings of this study are available from the corresponding author upon reasonable request.
